# How much attention is needed towards men who sell sex to men for HIV prevention in India?

**DOI:** 10.1186/1471-2458-6-31

**Published:** 2006-02-15

**Authors:** Lalit Dandona, Rakhi Dandona, G Anil Kumar, Juan Pablo Gutierrez, Sam McPherson, Stefano M Bertozzi

**Affiliations:** 1 Health Studies Area, Centre for Human Development, Administrative Staff College of India, Hyderabad, India; 2 Division of Health Economics and Policy, National Institute of Public Health, Cuernavaca, Mexico; 3 Research and Evaluation Unit, International HIV/AIDS Alliance, Brighton, UK

## Abstract

**Background:**

HIV prevention in India has mostly focussed on heterosexual transmission. Data on homosexual transmission are not readily available from India. We therefore assessed the probability of acquiring and transmitting HIV for men who sell sex to men and compared this with women who sell sex in India.

**Methods:**

Sexual behaviour characteristics of 6661 men who have sex with men and 6648 women who sell sex were obtained in the Indian state of Andhra Pradesh through confidential interviews. These, along with estimates of HIV rates among them and risk of HIV transmission per unprotected sex act from other sources, were used to calculate their annual probability of acquiring and transmitting HIV.

**Results:**

Of 6661 men who have sex with men in this sample, 1776 (26.7%) had sold sex to men. For every 1000 men who sell sex to men, annually 146 (95% confidence interval [CI] 116–179) would acquire HIV and HIV would be transmitted to 55 (95% CI 42–71) men who do not sell sex or women. These estimates were higher by 6.7 (95% CI 4.9–9.2) times for acquiring HIV and 2.5 (95% CI 2.0–3.2) times for transmitting HIV to sex partners outside their group, as compared with similar estimates for women who sell sex. In this sample, the average annual probability of acquiring HIV was higher among men who have sex with men but do not sell sex as compared with women who sell sex.

**Conclusion:**

These data indicate that men who sell sex to men are at much higher risk of acquiring and transmitting HIV than women who sell sex. Therefore, men who sell sex to men and their clients warrant substantial attention for comprehensive HIV prevention in India.

## Background

India is estimated to have one of the highest numbers of HIV-infected persons in the world [1]. The focus of HIV prevention in India has mostly been on heterosexual transmission, particularly that linked with women who sell sex [2]. As part of the Frontiers Prevention Project baseline study, we have recently reported that in a sample of 6661 men who have sex with men in the Indian state of Andhra Pradesh, over half had unprotected anal sex with men and a quarter had unprotected anal/vaginal sex with both men and women [3]. We have also reported that in a sample of 6648 women who sell sex in Andhra Pradesh about half had unprotected vaginal sex with their clients [4]. The Frontiers Prevention Project is based on the principle of providing a comprehensive package of interventions in geographically defined sites that are focused on population groups which are key to the dynamics of the HIV epidemic [3,4].

In this report we examine the relative probability of acquiring and transmitting HIV for men who sell sex to men and for women who sell sex in order to understand how much attention is needed towards men who sell sex to men for HIV prevention in India.

## Methods

Detailed data on sexual behaviour were collected in confidential interviews with 6661 men who have sex with men and 6648 women who sell sex from 40 sampled geographic sites covering urban and rural locations in 13 districts of the Indian state of Andhra Pradesh. This was done from July 2003 to April 2004 as part of the Frontiers Prevention Project baseline study, which was approved by the Ethics Committees of the Administrative Staff College of India, Mexico's National Institute of Public Health, the International HIV/AIDS Alliance, and by the Indian Health Ministry's Screening Committee, Indian Council of Medical Research, New Delhi, India.

Details of the sampling and data collection procedures for men who have sex with men and women who sell sex are described elsewhere [3,4]. Forty geographic sites in 13 districts of the Telangana and Rayalseema regions of Andhra Pradesh state were identified where men who have sex with men and women who sell sex were considered to be present in reasonably large numbers and access to them seemed feasible through peer facilitators and non-governmental organisations having links with them. Each geographic site consisted of one or more close-by cities/towns/villages. The total number of cities, towns or villages included in the 40 geographic sites were 62 for men who have sex with men and 72 for women who sell sex, which included a range of rural and urban categories of various sizes [3,4].

The data collection instruments were developed by a multidisciplinary team through review of worldwide literature, focus group discussions and in-depth interviews with men who have sex with men and women who sell sex for the local context in Andhra Pradesh, and pre-pilot studies. The instruments were translated into the local language Telugu, translation checked through back-translation into English, and refinement done where necessary. Extensive training of the interviewers was done by a variety of survey experts, as well as men who have sex with men and women who sell sex, in order to address the technical, ethical and cultural issues.

At each study location, peer facilitators helped contact and recruit respondents. Standardised procedures were established and followed for contacting and interviewing respondents. Written informed consent was obtained from each respondent. One-to-one interviews were done confidentially and the identity of respondents was not recorded. The methods used were similar for men who have sex with men and women who sell sex. Relevant to this report, the number of sex partners, number of times sex done in a unit time, type of sex done, condom use, whether men who have sex with men also sold sex to men, and if men sold sex to men was this done frequently or infrequently, were documented.

The probability of HIV infection was estimated using a previously published formula [5]: Pr = 1-{P [1-R(1-FE)]^N^+(1-P)}^M ^where Pr is the probability of HIV infection in uninfected, P is the average HIV prevalence among sex partners of the group for which the probability is being estimated, R is the risk of HIV transmission per act of unprotected sex, F is the fraction of sex acts in which condom is used, E is the effectiveness of condoms, N is the average number of sex acts per partner, and M is the average number of sex partners. In order to estimate the probability of new HIV infection in an entire group including those uninfected as well as infected by HIV, Pr was multiplied by (1 – I), where I is the proportion in the susceptible group that is already infected with HIV. Probabilities were calculated separately for acquiring and transmitting HIV. For the calculation of acquiring HIV, the application of the formula is self-explanatory. For the calculation of transmitting HIV, the group to which transmission was assessed became the acquirer in the formula, with the value of variables in the formula being those that were applicable to sex between these two groups. For example, while calculating transmission by men who sell sex to men of HIV to men who do not sell sex, the value for P was that for men who sell sex to men and the value for the other variables were those that applied to sex between these two groups.

The average HIV prevalence was 16% among men who have sex with men and 16% among women who sell sex in the sentinel surveillance of 2004 in Andhra Pradesh [6]. As the sample in our study was recruited through peers and non-governmental organisations, it was felt that the HIV risk characteristics were likely to be similar in our sample and the sample recruited through non-governmental organisations in the sentinel surveillance. Therefore, HIV prevalence from the sentinel surveillance was used for our sample. Among men who have sex with men, HIV prevalence was assumed to be higher for those who sell sex to men (24%) than in those who do not sell sex to men (12%). Among men who sell sex to men, HIV prevalence was assumed to be higher for those who sell sex frequently (25%) as compared with those who sell sex infrequently (22%). When sex partners of men who have sex with men formed a mix of those who sold sex and those who did not, HIV prevalence intermediate to those for these groups was used for calculations based on the proportions – of the men who have sex with men who do not sell sex 17.3% paid for sex and of the men who sell sex and also do insertive anal sex 30.3% paid for sex in our sample. HIV prevalence among clients of women who sell sex was assumed to be half that in women who sell sex (8%). HIV prevalence among women sex partners of men who have sex with men assumed to be 4% – about twice the estimate for general women in the antenatal sentinel surveillance of 2004 in Andhra Pradesh [6].

The risk of HIV transmission per unprotected sex act was adapted from previous literature [7-9]. As probabilities for acquiring HIV per unprotected sex act are estimated to be higher in developing countries due to co-existing sexually transmitted infections and other factors [9], among the reported ranges relatively higher values were assumed for our sample: 0.015 for receptive anal sex, 0.001 for insertive anal sex, 0.0015 for receptive vaginal sex, 0.0007 for insertive vaginal sex, and 0.0004 for receptive oral sex.

Estimation of the effectiveness of condoms was based on a Cochrane systematic review, which reported that the effectiveness of condom in reducing HIV transmission during vaginal sex was 0.80 [10]. Since condom use is less effective for anal sex than for vaginal sex, especially without lubricant use as in our setting [3], we assumed a lower condom effectiveness of 0.70 for anal sex. On the other hand, since condom use is likely to be more effective for oral sex than for vaginal sex, we assumed a higher condom effectiveness of 0.90 for oral sex. It is to be noted that this effectiveness is in the real life situation, as compared with condom efficacy in an ideal situation which would be higher.

The following range of values were considered plausible for the variables in the formula: 20% lower and higher than the point estimates for P, R and I used in the base calculations; the low and high values of the 95% confidence intervals for the estimates of F, N and M in our data; E for anal sex 0.65–0.75, for vaginal sex 0.75–0.85 and for oral sex 0.85–0.95. Due to limited availability of published data from India, the plausible ranges assumed by us were based on what seemed reasonable to us. A 20% variation on either side of the P, R and I estimates and 0.05 variation on either side of risk per unprotected anal, vaginal or oral sex seemed reasonable plausible ranges. For F, N and M, variables for which we had data from our field study, the 95% confidence intervals were considered as the plausible range. Sensitivity analysis for the probabilities of acquiring and transmitting HIV was performed based on the Monte Carlo simulation principle by doing 100,000 iterations with the @Risk software (Palisade Corporation, Newfield, New York, USA), using random values between these plausible ranges to obtain the range and 95% confidence intervals for the estimates for the probabilities of acquiring and transmitting HIV. The @Risk software was also used to assess the sensitivity of the probability ratios between men who sell sex and women who sell sex to the different variables in the formula.

## Results

In an earlier publication, we mentioned that of the 6661 men who have sex with men in our sample, 609 (9.1%) reported that they were sex workers [3]. However, as compared with this self-reporting as sex workers, probing through other questions whether they had received money for sex revealed that that a much higher number [1776 (26.7%)] had sold sex to men. Of these 1776, 1098 (61.8%) reported that they sold sex to men frequently and 678 (38.2%) reported that they sold sex to men infrequently. Of the 1098 men who reported selling sex to men frequently, 553 (50.4%) self-reported their primary or secondary occupation as sex worker, whereas of the 678 men who reported selling sex to men infrequently only 56 (8.3%) did so. The age distribution was not significantly different between men who sold sex to men and those who did not (p = 0.38, analysis of variance), or between men who sold sex to men frequently and those who did so infrequently (p = 0.92, analysis of variance) (Table 1). Of the total men who have sex with men in our sample from the various rural and urban categories, those who sold sex to men were found in all categories with the proportions ranging from 21.1% to 41.4%, the highest being for the sample from the largest cities (Table 2).

The base calculations for the annual probability of acquiring and transmitting HIV for men having sex with men who sell sex and who do not sell sex, and for women who sell sex, are shown in Table 3. For every 1000 men who sell sex to men, in a year 146 would acquire HIV and HIV would be transmitted to 43 men who do not sell sex and to 12 women. This was 6.7 times higher for acquiring HIV and 2.5 times higher for transmitting HIV to sex partners outside their group as compared with the respective probabilities for women who sell sex.

Sensitivity analysis using random values of the variables in the formula within their plausible extremes revealed that the 95% confidence interval for the higher probability of men who sell sex to men as compared with women selling sex was 4.9 to 9.2 times for acquiring HIV and 2.0 to 3.2 times for transmitting HIV outside their group (Table 4). For acquiring HIV these results were most sensitive to the average HIV prevalence among sex partners and the risk of HIV transmission per act of unprotected sex for receptive vaginal and receptive anal sex, and for transmitting HIV the results were most sensitive to the average HIV prevalence among sex partners and the risk of HIV transmission per act of unprotected sex for insertive vaginal sex and to a lesser degree to these same variables for insertive and receptive anal sex (Table 4).

For the men who reported selling sex frequently the average probability of acquiring HIV was twice, of transmitting HIV to men who do not sell sex slightly higher, and of transmitting HIV to women half, as compared with those who reported selling sex infrequently (Table 3). A man who sold sex to men had 2.4 times higher probability of acquiring HIV and 2.8 times higher probability of transmitting HIV to sex partners outside his group, as compared with a man who had sex with men but did not sell sex. In this sample, a man who had sex with men but did not sell sex had a 2.7 times higher probability of acquiring HIV, and a slightly lower probability of transmitting HIV outside his group, as compared with women who sold sex.

## Discussion

There are recent reports regarding various aspects of the HIV risk of men who sell sex to men in some parts of the world [12-16], but such data from India are not readily available. Although our estimates in this report should be considered only indicative, these suggest that the average probability of acquiring or transmitting HIV for a man who sells sex to men is many-fold that of a woman who sells sex in this Indian state. The annual 11.6–17.9% chance of acquiring HIV among men who sell sex to men is strikingly higher than the 1.7–2.7% chance for women who sell sex in our sample. In addition, the number of persons outside their group to whom HIV is likely transmitted was two and a half times higher for each man who sells sex to men as compared with a woman who sells sex. Good estimates for the total number of men who sell sex to men are not available for Andhra Pradesh or for other parts of India. Although the total number of women who sell sex is likely to be much higher than men who sell sex to men in Andhra Pradesh, the number of men who sell sex to men does not seem to be negligible, going by the substantial number who participated in our study. The background of sex between men being a very hidden entity in India makes this substantial number of men who sell sex to men in our sample even more significant. The much higher average HIV risk of men who sell sex to men as compared with women who sell sex, and the likely presence of a sizeable number of men who sell sex to men, indicate that they and their clients too need urgent and systematic attention in HIV prevention efforts in India. This higher HIV risk of men who sell sex to men is largely due to the approximately ten-fold higher risk of transmission of HIV per act of unprotected receptive anal sex as compared with unprotected receptive vaginal sex. It should be noted though that the annual risks of acquiring and transmitting HIV presented in this paper are based on the current estimates of the variables that go into this calculation. These annual risks would change as the estimates for the variables change over time, for example if the HIV prevalence changes or prevention efforts lead to change in the average number of sex partners and/or condom use.

In our sample the men who have sex with men but did not sell sex also had a higher probability of acquiring HIV as compared with women who sell sex. However, we believe that our sample is not representative of all men who have sex with men in Andhra Pradesh, as we accessed them through peers and non-governmental organisations working with them, resulting in our getting relatively more visible and sexually active men who have sex with men in our sample. Men who have sex with men are not openly accepted in the Indian society due to social taboo. Since the men who sell sex to men have an estimated two and a half to three times higher probability of acquiring HIV and of transmitting it outside their group as compared with the men who have sex with men but do not sell sex, and because they are potentially more accessible than the later, it would seem reasonable to build up HIV prevention related to men who have sex with men in India by initially focusing on the men who sell sex and their clients. However, it should be noted that many men who sell sex to men in India would not be easily accessible either, as suggested by our data that only half of the men who sold sex to men frequently, and only 8% of those who sold sex to men infrequently in our sample, identified themselves as sex workers. Of course, the men who have sex with men but do not sell sex to men or buy sex from men would also have to be covered gradually by the HIV prevention efforts. This would be facilitated if the Indian society develops a more neutral stance towards men who have sex with men that allows them to openly acknowledge their sexual behaviour, which would enable them to access HIV prevention without fear or discrimination.

The data for HIV prevalence among men who have sex with men and women who sell sex, transmission risk of each unprotected sex act, and effectiveness of condom used by us were adapted from other sources, which is a limitation of the calculations presented in this paper. As explained in the methods section, we used the estimates and their plausible ranges that seemed to be most reasonable for our setting. On the other hand, the use of detailed sex behaviour data from very large samples of men who have sex with men, including men who sell sex to men, and of women who sell sex is a strength of the HIV probability calculations that we have presented.

The findings presented in this report have implications for a balanced use of the large resources that are increasingly becoming available for HIV prevention in India. If HIV prevention efforts in India have to be optimally effective, no groups at substantial risk of HIV can be overlooked, including men who sell sex to men and their clients. From the relatively large number of men who sell sex to men in our sample it seems that their numbers may not be too small, but more thorough assessment of this denominator would be useful for informed planning of HIV prevention in India.

## Conclusion

These data, based on detailed sex behaviour in very large samples of men who have sex with men and women who sell sex in Andhra Pradesh state of India, show a much higher probability of acquiring HIV for men who sell sex to men than for women who sell sex and a higher probability of transmitting HIV to sex partners outside their group. This suggests that men who sell sex to men and their clients also warrant substantial attention for comprehensive HIV prevention in India. It would therefore be useful for policy makers to keep in mind that HIV prevention programmes for males who sell sex to males need to be an important component of the HIV prevention effort in India.

## Competing interests

The authors declare that they have no competing interests.

## Authors' contributions

LD conceived the idea of this report, contributed to the design of the Frontiers Prevention Project baseline study, designed and led the analysis and interpretation for this report, and drafted the manuscript. RD contributed to the idea of this report and the design of the Frontiers Prevention Project baseline study, and to the design, analysis and interpretation for this report. GAK contributed to the data management, statistical analysis and interpretation. JPG and SMB contributed to the design of the Frontiers Prevention Project baseline study, and to the analysis and interpretation for this report. SM contributed to the design of the Frontiers Prevention Project baseline study and to the interpretation for this report. All named authors read, commented on and approved the final version of the manuscript. The ASCI FPP Study Team contributed to the planning of the Frontiers Prevention Project baseline study logistics, data collection and interpretation, and the members of this Team other than the named authors include (in alphabetical order): G Md Mushtaq Ahmed, Md Akbar, Md Abdul Ameer, Ch Arjun, N Arjun, M Sai Baba, C Satish Babu, J Kishore Babu, I Balasubrahmanyam, V S Udaya Bhaskar, T Gangadhar, P Gopal, Lavanya Gotety, Shaik Omar Hussain, V Indira, S Krishna, P Kiran Kumar, Ch Sri Jaya Lakshmi, T Uma Maheshwar, P Chandra Mouli, S Radhakrishnan, K Raghu, S P Ramgopal, A Srinivas Rao, A Srinivasa Rao, K Hanumantha Rao, N Ananda Rao, P Venkateswara Rao, Parsa V R Rao, D Ravinder, A Srinivas Reddy, G Brahmananda Reddy, S Krishna Reddy, G Uma Sankar, A Satyam, Y S Sivan, P V Sridhar.

## Pre-publication history

The pre-publication history for this paper can be accessed here:



## Figures and Tables

**Table 1:** Age distribution of men who sell sex to men and men who have sex with men but do not sell sex

**Age **(years)	**Men who had sex with men but did not sell sex ** Number (%)*	**Men who sold sex to men ** Number (%)*	**Men who sold sex to men frequently ** Number (%)*	**Men who sold sex to men infrequently ** Number (%)*
16–19	342 (7.0)	128 (7.2)	74 (6.7)	54 (8.0)
20–24	1309 (26.8)	536 (30.2)	329 (30.0)	207 (30.5)
25–29	1476 (30.2)	496 (27.9)	314 (28.6)	182 (26.8)
30–34	817 (16.7)	274 (15.4)	176 (16.0)	98 (14.5)
35–39	519 (10.6)	173 (9.7)	109 (9.9)	64 (9.4)
40–44	255 (5.2)	96 (5.4)	52 (4.7)	44 (6.5)
45 or more	167 (3.4)	73 (4.1)	44 (4.0)	29 (4.3)
Total	4885 (100)	1776 (100)	1098 (100)	678 (100)

*Percent of the total for each column.

**Table 2:** Proportion of men who sell sex among the sample of men who have sex with men in various rural-urban categories

Rural-urban category* (population)	**Number of villages/ towns/cities**	**Total number in sample**	**Men who sold sex to men **Number (%)†	**Men who sold sex to men frequently **Number (%)†	**Men who sold sex to men infrequently **Number (%)†
Rural	18	965	306 (31.7)	208 (21.6)	98 (10.2)
Urban (50,000 or less)	9	502	137 (27.3)	91 (18.1)	46 (9.2)
Urban (50,001–100,000)	15	1511	326 (21.6)	157 (10.4)	169 (11.2)
Urban (100,001–200,000)	10	1651	405 (24.5)	266 (16.1)	139 (8.4)
Urban (200,001–300,000)	6	1180	249 (21.1)	139 (11.8)	110 (9.3)
Urban (>300,000)	4	852	353 (41.4)	237 (27.8)	116 (13.6)
Total	62	6661	1776 (26.6)	1098 (16.5)	678 (10.2)

*Rural-urban definition and the populations taken from the Census of India [11].

†Percent of the total number in each rural-urban category.

**Table 3:** Annual probability of acquiring and transmitting HIV for men having sex with men who sell sex and who do not sell sex and for women who sell sex

**Men who sell sex to men **(N = 1776)									
Variable*	**For acquiring HIV through sex with men**			**For transmitting HIV through sex with women**			**For transmitting HIV through sex with men who do not sell sex**		
	
	Receptive anal sex	Receptive oral sex	Insertive anal sex	Receptive vaginal sex	Receptive anal sex	Receptive oral sex	Insertive anal sex	Receptive anal sex	Receptive oral sex

P	0.13	0.13	0.16	0.24	0.24	0.24	0.24	0.24	0.24
R	0.015	0.0004	0.001	0.0015	0.015	0.0004	0.001	0.015	0.0004
F	0.53	0.35	0.53	0.11	0.33	0.13	0.53	0.53	0.12
E	0.70	0.90	0.70	0.80	0.70	0.90	0.70	0.70	0.90
N	3.09	1.59	3.09	29.69	29.69	29.69	3.09	3.09	3.31
M	55.81	19.86	4.97	1.02	0.03	0.07	54.30	3.46	1.42
I	0.24	0.24	0.24	0.04	0.04	0.04	0.12	0.12	0.12
Annual probability per 1000	143.86	0.85	1.17	9.34	2.09	0.17	22.00	20.94	0.35

Total	146			12			43		

**Men who sell sex to men frequently **(N = 1098)									

									
Variable*	**For acquiring HIV through sex with men**			**For transmitting HIV through sex with women**			**For transmitting HIV through sex with men who do not sell sex**		
	
	Receptive anal sex	Receptive oral sex	Insertive anal sex	Receptive vaginal sex	Receptive anal sex	Receptive oral sex	Insertive anal sex	Receptive anal sex	Receptive oral sex

P	0.13	0.13	0.16	0.25	0.25	0.25	0.25	0.25	0.25
R	0.015	0.0004	0.001	0.0015	0.015	0.0004	0.001	0.015	0.0004
F	0.57	0.38	0.57	0.122	0.40	0.222	0.57	0.57	0.12
E	0.70	0.90	0.70	0.80	0.70	0.90	0.70	0.70	0.90
N	2.85	1.56	2.85	29.14	29.14	29.14	2.85	2.85	3.31
M	76.89	27.02	4.34	0.74	0.02	0.03	75.50	2.95	1.21
I	0.25	0.25	0.25	0.04	0.04	0.04	0.12	0.12	0.12
Annual probability per 1000	168.87	1.08	0.89	6.88	1.35	0.07	27.98	16.42	0.31

Total	171			8			45		

**Men who sell sex to men infrequently **(N = 678)									

Variable*	**For acquiring HIV through sex with men**			**For transmitting HIV through sex with women**			**For transmitting HIV through sex with men who do not sell sex**		
	
	Receptive anal sex	Receptive oral sex	Insertive anal sex	Receptive vaginal sex	Receptive anal sex	Receptive oral sex	Insertive anal sex	Receptive anal sex	Receptive oral sex

P	0.13	0.13	0.16	0.22	0.22	0.22	0.22	0.22	0.22
R	0.015	0.0004	0.001	0.0015	0.015	0.0004	0.001	0.015	0.0004
F	0.47	0.27	0.47	0.10	0.30	0.09	0.47	0.47	0.12
E	0.70	0.90	0.70	0.80	0.70	0.90	0.70	0.70	0.90
N	4.00	1.94	4.00	30.00	30.00	30.00	4.00	4.00	3.31
M	23.07	8.92	5.22	1.48	0.05	0.13	21.57	3.72	1.52
I	0.22	0.22	0.22	0.04	0.04	0.04	0.12	0.12	0.12
Annual probability per 1000	87.68	0.53	1.75	12.61	3.28	0.30	11.13	28.24	0.35

Total	90			16			40		

**Men who have sex with men but do not sell sex **(N = 4885)									

Variable*	**For acquiring HIV through sex with men**			**For transmitting HIV through sex with women**			**For transmitting HIV through sex with men who sell sex**		
	
	Receptive anal sex	Receptive oral sex	Insertive anal sex	Receptive vaginal sex	Receptive anal sex	Receptive oral sex	Insertive anal sex	Receptive anal sex	Receptive oral sex

P	0.16	0.16	0.18	0.12	0.12	0.12	0.12	0.12	0.12
R	0.015	0.0004	0.001	0.0015	0.015	0.0004	0.001	0.015	0.0004
F	0.51	0.12	0.51	0.16	0.22	0.10	0.57	0.53	0.35
E	0.70	0.90	0.70	0.80	0.70	0.90	0.70	0.70	0.90
N	3.89	3.31	3.89	34.09	34.09	34.09	2.85	3.09	1.59
M	10.64	4.35	13.13	2.26	0.05	0.05	2.95	2.27	0.81
I	0.12	0.12	0.12	0.04	0.04	0.04	0.24	0.24	0.24
Annual probability per 1000	53.87	1.24	5.18	11.31	2.08	0.07	0.46	5.97	0.03

Total	60			13			6		

**Women who sell sex **(N = 6648)									

Variable*	**For acquiring HIV through sex with men**			**For transmitting HIV through sex with men**					
	
	Receptive vaginal sex	Receptive anal sex	Receptive oral sex	Insertive vaginal sex	Insertive anal sex				
P	0.08	0.08	0.08	0.16	0.16				
R	0.0015	0.015	0.0004	0.0007	0.001				
F	0.63	0.59	0	0.63	0.59				
E	0.80	0.70	0.90	0.80	0.70				
N	2.51	2.51	2.51	2.51	2.51				
M	171.78	0.17	0.43	171.78	0.17				
I	0.16	0.16	0.16	0.08	0.08				
Annual probability per 1000	21.27	0.25	0.03	21.77	0.04				
Total	22			22					

*P is average HIV prevalence among sex partners of the group for which the probability is being estimated; R is risk of HIV transmission per act of unprotected sex; F is fraction of sex acts in which condom was used; E is effectiveness of condoms; N is average number of sex acts per partner in a year; M is average number of sex partners in a year; I is proportion of the susceptible group that is already infected with HIV.

**Table 4:** Sensitivity analysis for relative probabilities between men who sell sex to men and women who sell sex for acquiring and transmitting HIV

	**Annual probability per 1000**					
	
	Men who sell sex to men		Women who sell sex		Ratio between men who sell sex to men and women who sell sex†	
	
	95% confidence interval*	Range*	95% confidence interval*	Range*	95% confidence interval*	Range*
**Total for acquiring HIV through sex with men**	116 – 179	94 – 211	17 – 27	14 – 32	4.9 – 9.2	3.7 – 12.8
**Total for transmitting HIV to outside their group**	42 – 71	33 – 86	18 – 26	15 – 30	2.0 – 3.2	1.5 – 4.3
For transmitting HIV through sex with women	8 – 16	6 – 20				
For transmitting HIV through sex with men who do not sell sex	34 – 55	27 – 66				
For transmitting HIV through sex with men			18 – 26	15 – 30		

*Calculated with @Risk software from 100,000 iterations using random values between the plausible range of values for the variables in the formula. The range of values considered plausible is explained in the methods section.

†For acquiring HIV, this ratio was most sensitive to P for receptive vaginal sex (-0.50), R for receptive vaginal sex (-0.50), P for receptive anal sex (0.45) and R for receptive anal sex (0.44); for transmitting HIV, the results were most sensitive to P for insertive vaginal sex (-0.64), R for insertive vaginal sex (-0.45), and then to P and R for insertive anal sex (0.25 for both) and P and R for receptive anal sex (0.24 for both). The sensitivity of this ratio to the plausible range of values used for each variable can range from 0 (no sensitivity) to -1 (complete negative sensitivity) or 1 (complete positive sensitivity). Letters denoting the variables are explained in the methods section and the footnote for Table 3.
